# The Deformities of the Human Foot

**Published:** 1895-12

**Authors:** 


*The Deformities of the Human Foot.* By W. J. WALSHAM, M.B., C.M., and WILLIAM KENT HUGHES, M.B. Pp. vi., 550. London: BAILLIÈRE, TINDALL AND COX. 1895.

This work is mainly a record of thirteen years experience by Mr. Walsham in the Orthopædic Department at St. Bartholomew's Hospital, in which some seven hundred to eight hundred patients are treated annually. He has been assisted by Mr.

Kent Hughes, who was formerly his senior dresser in the department; the latter having taken numerous photographs from patients and from casts and specimens in the museum. The result is a useful and practical manual, containing a great deal of valuable information on a very important subject.

The greatest part of the book is naturally devoted to talipes, and no less than a fourth of the whole treats of one variety of talipes, viz., congenital talipes varus. As regards etiology, four theories are alluded to, viz.: (1) the spasmodic muscular contraction theory; (2) the mechanical theory; (3) the arrest of development theory; (4) the defect-in-the-germ theory. Of these the authors consider that the mechanical is the most tenable, and we think they give very good reasons for their opinion. Much space is devoted to the treatment of this affection; and the various mechanical appliances are fully and plainly described. In the treatment of the more severe forms of the deformity, various cutting operations are described, including Phelps's method by open incision on the inner side, and the numerous tarsotomies and tarsectomies, which have been advocated by others; but we are glad to notice that the authors rely mainly upon conservative measures for the treatment of all but a few inveterate cases.

English authors have been freely drawn upon throughout the work; but very little notice is taken of foreign workers. One or two French writers are alluded to, but German literature is apparently ignored. The result is that the work is by no means complete as a book of reference, and cannot be relied upon as a scientific attempt to deal with the subject; but it will serve a useful purpose as a practical guide to modern diagnosis and treatment.

